# Multi-wavelength photoacoustic imaging of inducible tyrosinase reporter gene expression in xenograft tumors

**DOI:** 10.1038/srep05329

**Published:** 2014-06-17

**Authors:** Robert J. Paproski, Andrew Heinmiller, Keith Wachowicz, Roger J. Zemp

**Affiliations:** 1 Department of Electrical and Computer Engineering, University of Alberta, Edmonton, Alberta T6G 2V4, Canada; 2 FUJIFILM VisualSonics, Inc., Toronto, Ontario M4N 3N1, Canada; 3 Department of Oncology, University of Alberta, Edmonton, Alberta T6G 1Z2, Canada

## Abstract

Photoacoustic imaging is an emerging hybrid imaging technology capable of breaking through resolution limits of pure optical imaging technologies imposed by optical-scattering to provide fine-resolution optical contrast information in deep tissues. We demonstrate the ability of multi-wavelength photoacoustic imaging to estimate relative gene expression distributions using an inducible expression system and co-register images with hemoglobin oxygen saturation estimates and micro-ultrasound data. Tyrosinase, the rate-limiting enzyme in melanin production, is used as a reporter gene owing to its strong optical absorption and enzymatic amplification mechanism. Tetracycline-inducible melanin expression is turned on via doxycycline treatment in vivo. Serial multi-wavelength imaging reveals very low estimated melanin expression in tumors prior to doxycycline treatment or in tumors with no tyrosinase gene present, but strong signals after melanin induction in tumors tagged with the tyrosinase reporter. The combination of new inducible reporters and high-resolution photoacoustic and micro-ultrasound technology is poised to bring a new dimension to the study of gene expression in vivo.

Reporter genes can help elucidate gene expression signatures in vivo^1^. When expressed in cells, reporter genes produce contrast in an imaging modality. Reporter genes have long been used in positron emission tomography^2^, fluorescence imaging modalities^3^, bioluminescence systems^4^, magnetic resonance imaging^5^, and more recently ultrasound and photoacoustic imaging^6^,^7^. Optical platforms are particularly attractive owing to their relative simplicity for pre-clinical studies. However, optical imaging technologies struggle to produce high-resolution images at significant depths due to optical scattering.

Photoacoustic imaging, also known as optoacoustic imaging, offers an alternative to optical-scattering limited modalities^8^,^9^. A short-pulsed laser causes localized heating in optically absorbing materials, producing pressure waves. These pressure waves are measured with an ultrasound transducer and provide ultrasonic-resolution imaging of optical absorption, allowing for deep, non-invasive, high optical contrast and high spatial-resolution imaging using non-ionizing radiation.

Suitable optical reporter genes for photoacoustic imaging are beginning to emerge. While lacZ (which encodes β-galactosidase) has been used as a reporter gene for photoacoustic imaging^6^,^10^,^11^, it depends on the metabolism of X-gal (5-bromo-4-chloro-indolyl-β-D-galactoside) which can cause skin irritation, and, in rodent models, can be difficult to deliver to xenograft tumors through tail-vein injection^6^. Fluorescent proteins have recently been investigated for photoacoustic molecular imaging^12^,^13^,^14^. Multi-wavelength techniques are typically required to estimate fluorophore distributions^12^,^14^. Although Razansky et al. was capable of visualizing tissue-specific eGFP and mCherry fluorescent protein expression in *Drosophila melanogaster* pupa and zebrafish, respectively, using photoacoustic imaging, there are some disadvantages of using fluorescent protein cDNA as reporter genes for photoacoustic imaging^15^.

Fluorescent proteins are typically designed for high fluorescent yield, meaning the fraction of energy converted to heat for photoacoustic signal generation is not as high as might be possible with a low-quantum yield species. Finally most fluorescent proteins do not have the amplification potential of enzymatic reporter genes. For an enzymatic reporter gene, one molecule of the enzyme can interact with many substrate molecules, thus providing signal amplification. Finally, fluorescent proteins are prone to laser-induced absorption bleaching, complicating photoacoustic imaging.

Tyrosinase is an exciting potential enzymatic reporter gene as it converts tyrosine to dopaquinone: the rate-limiting step in the production of melanin^16^. Melanin offers strong optical absorption in the visible and near infrared spectrum^17^. One tyrosinase protein can potentially catalyze many melanin sub-units, providing an amplification strategy. Tyrosinase has been shown to be a promising optical and photoacoustic reporter gene in studies by our group in vitro^7^ as well as by Krumholz et al.^18^ using optical resolution photoacoustic microscopy (OR-PAM, a photoacoustic technique depending on laser focusing to provide high resolution images at a shallow <1 mm depth). Laufer demonstrated imaging of melanin-expressing tumor cells using a Fabry-Perot optoacoustic approach^19^. Qin et al.^20^ showed that tyrosinase could be used as a triple-modality reporter for positron emission tomography, MRI, and optoacoustic imaging in vivo. While highly promising results were demonstrated, this work did not demonstrate inducible expression and used only a single wavelength for photoacoustic imaging, precluding spectral unmixing approaches to decouple melanin from blood absorption. Stritzker et al.^21^ introduced oncolytic viral-delivery of tyrosinase as a theranostic agent for MRI, optoacoustic imaging, and photothermal therapy. They were able to visualize metastases with multispectral optoacoustic tomography but without quantifying false positive levels or the extent of in vivo transfection to non-tumor tissues. In vivo viral delivery approaches are not always suitable and require significant precautions. Transduction of genes in mammalian cells by some viruses (especially oncolytic viruses^22^) can greatly alter the biology (growth rates, signalling pathways, etc^23^,^24^) of mammalian cells which may not be desirable when these viral-induced cellular alterations are not normally present in the model system being studied. The Stritzker et al.^21^ approach was designed to be therapeutic as well as diagnostic, but given the unknown effects of melanin production on tumor growth kinetics and the lytic effects of the oncolytic virus, it was unclear whether the approach could be diagnostic without being therapeutic as one might wish for in a longitudinal study of tumor progression or non-oncologic studies of in vivo gene regulation.

Inducible systems represent an important class of reporter technologies, where the gene of interest (in this case tyrosinase) is not turned on (or off) until an inducer such as doxycycline (DOX) is added. DOX treatment can be administered in vivo by adding it to the drinking water of animals. Inducible systems permit mitigation of the effects of the reporter on normal development and have been used for a broad range of pre-clinical applications^25^,^26^,^27^. After turning on melanin production, tissues with tyrosinase-expressing cells may be visible with photoacoustic imaging, MRI, PET, and scintigraphy, as previously reported. Inducible melanin expression for MRI was previously reported in vitro^7^. Here our focus is on multiwavelength photoacoustic imaging. Not only could this be important for studying tumor gene regulation in vivo and in response to various treatment regimens, but it could also be important for tracking metastases and tumor progression in response to therapeutics. There is also a great need for locating tumor micro-metastases for further imaging studies or histopathological studies, a task which is non-trivial. The research community is significantly lacking low-cost imaging tools for locating and enumerating such metastases in vivo and photoacoustic imaging may play an important role. Beyond oncologic imaging studies, inducible photoacoustic reporter systems could prove highly valuable in other pre-clinical arenas including development of transgenic and knockout models for neuroscience and developmental biology, among other applications.

In this paper we demonstrate inducible melanin production as a reporter strategy for in vivo high-resolution multi-wavelength photoacoustic imaging and MRI. We perform constrained spectral unmixing to estimate not only the relative melanin distribution, but also relative hemoglobin oxygen saturation. Volumetric photoacoustic imaging co-registered with micro-ultrasound is moreover demonstrated. We used a Tet-On® inducible tyrosinase reporter-gene system which permits normal cell behaviors until DOX induces tyrosinase expression and melanin production to enable photoacoustic visualization of in vivo gene expression. To our knowledge this is the first report of an inducible in vivo reporter gene system visualized by photoacoustic imaging, and the first report of multi-spectral photoacoustic unmixing to estimate relative gene expression and hemoglobin oxygen saturation in vivo. Our results show highly promising results with the ability to detect strong melanin-induced optical absorption in xenograft tumors with high spatial resolution and co-registration with high-frequency ultrasound.

## Results

### Inducible melanin production in cultured cell lines and xenograft tumors

**Figure 1:**
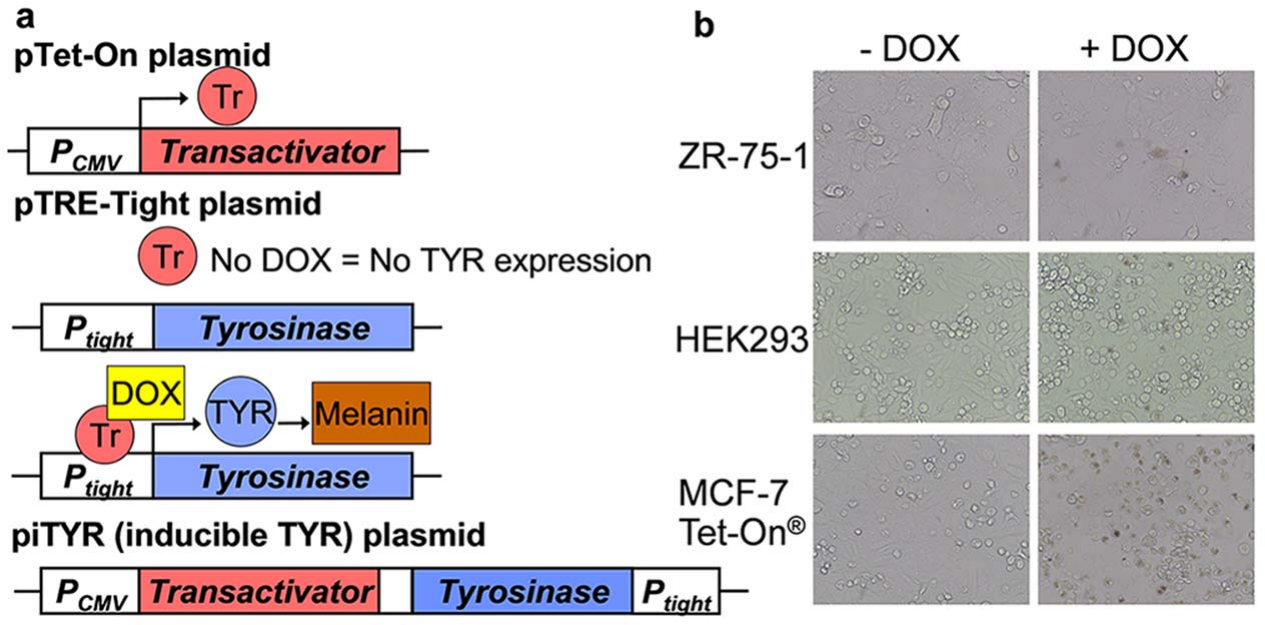
Inducible expression of tyrosinase using the Tet-On® system. (a) Tet-On® inducible system involves a transactivator gene under the expression of the constitutive CMV promoter (MCF-7 Tet-On® cells stably express the transactivator). Tyrosinase cDNA was inserted downstream of the *P*tight promoter on the pTRE-Tight plasmid which was stably transfected in MCF-7 Tet-On® cells (called +TYR cells). The transactivator is a transcription factor which, only in the presence of DOX, induces transcription at the *P*tight promoter causing tyrosinase expression and melanin production. piTYR plasmid contains the entire DOX inducible system on one plasmid. (b) Transient transfection of piTYR plasmid into ZR-75-1, HEK293, and MCF-7 Tet-On® cells allows DOX inducible expression of tyrosinase causing visible melanin production.

Two approaches were taken to transfect tumor cell lines to incorporate an inducible tyrosinase reporter system. The first approach is similar to Alfke et al.^28^, except we stably transfected MCF-7 Tet-On® cells with a pTRE-Tight vector containing the tyrosinase gene, creating +TYR cells (Fig. 1a). MCF-7 Tet-On cells without transfection of tyrosinase were called -TYR cells. Tyrosinase expression in the pTRE-Tight plasmid requires a Tet-On cell line for tyrosinase expression. However, not all cell lines of interest are commercially available with Tet-On capabilities. To address this, we also developed a single plasmid piTYR (Fig. 1a) containing both the constitutively expressed DOX responsive transactivator as well as the tyrosinase gene under the regulation of the *P*_tight_ promoter. MCF-7 Tet-On, ZR-75-1, and HEK293 cells were transiently transfected with piTYR using lipofectamine 2000 and all cell lines showed melanin production when cultured with 1 µg/mL DOX for three days (Fig. 1b). In both the multi-step (pTet-ON + pTRE-Tight-TYR) as well as single-plasmid approach (piTYR) we used the constitutively-active CMV promoter for transactivator expression although future work could instead use a different gene-specific promoter.

Photoacoustic imaging was performed on tubes containing -TYR and +TYR cells cultured with or without DOX as well as a tube of blood. Only tubes containing blood and +TYR cells cultured with DOX provided significant photoacoustic signal (Supplementary Fig. 1). Using the multispectral unmixing algorithm, photoacoustic signal from +TYR cells could be resolved from that of blood, suggesting that our unmixing method should allow discrimination of blood and tyrosinase-expressing cells in animal imaging.

**Figure 2:**
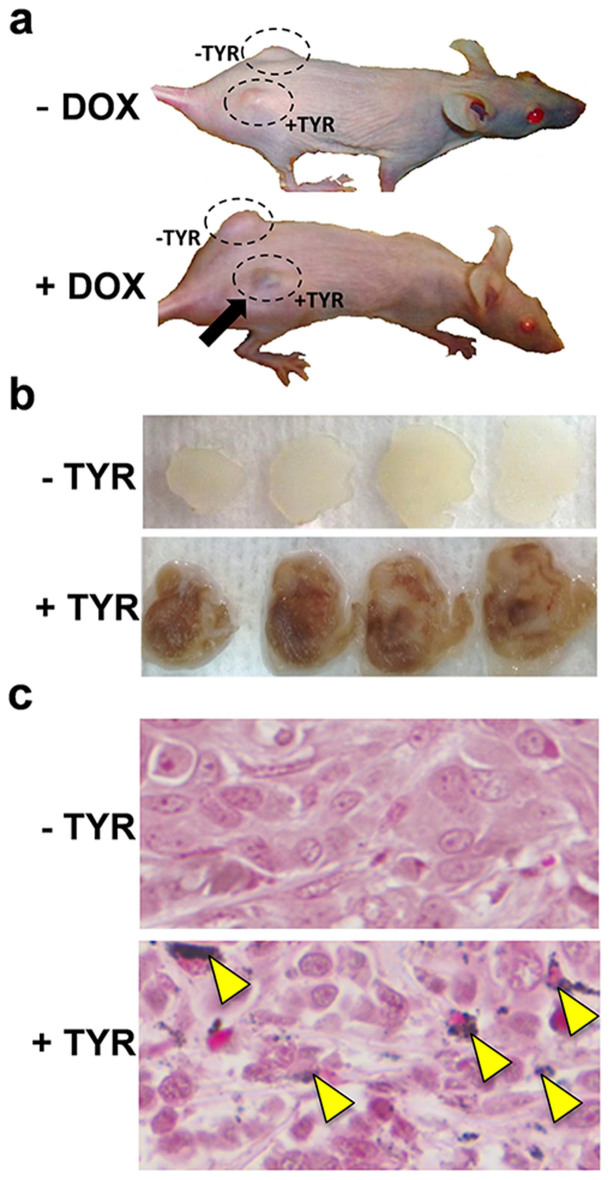
In vivo inducible tyrosinase expression in xenograft MCF-7 tumors. (a) Photographs of a mouse bearing –TYR and +TYR tumors before and after DOX administration. (b) Photographs of excised xenograft -TYR and +TYR tumors after 1 week of DOX treatment. (c) -TYR and +TYR tumor sections with Fontana Masson staining detecting melanin granules (yellow arrows).

-TYR and +TYR cells were injected subcutaneously in the back left and right flanks, respectively, of hairless SCID mice. When tumors were at least 1 mm in diameter, mice were given 1 mg/mL DOX drinking water to induce tyrosinase expression. One week after tyrosinase induction with DOX, +TYR tumors were visually darker than -TYR tumors as determined by eye (Fig. 2a) and half mm tumor sections (Fig. 2b), suggesting that melanin was produced in +TYR tumors. Dark pigment was heterogeneous throughout the +TYR tumors. Fontana-Mason staining of 8 µm tumor sections confirmed melanin granules exclusively within cells of the +TYR tumors (Fig 2c), suggesting that tyrosinase expression was capable of producing melanin in vivo with DOX induction.

### In vivo multiwavelength photoacoustic imaging studies

**Figure 3:**
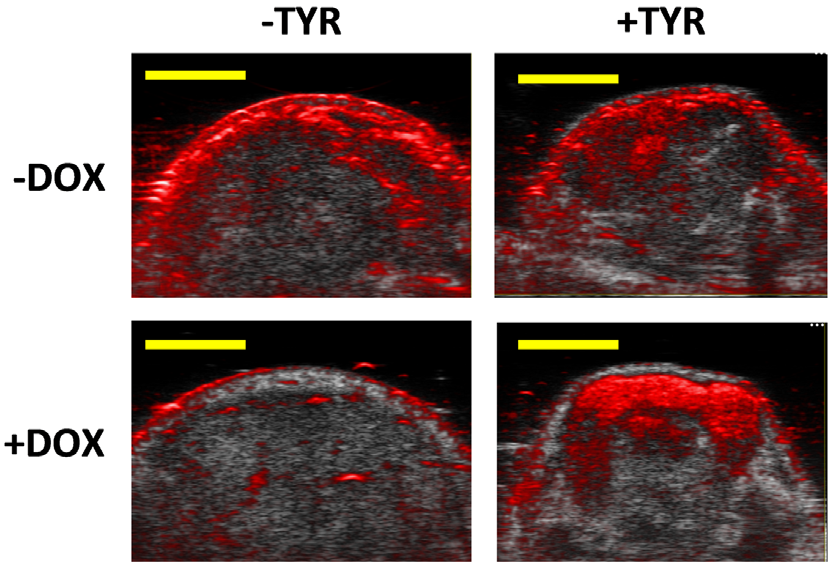
Photoacoustic (680 nm) and micro-ultrasound (grayscale) co-registered imaging of -TYR and +TYR tumors with and without DOX treatment. Scale bars represent 2 mm.

-TYR and +TYR tumors from six mice were imaged before and 1 week after DOX treatment using a VisualSonics Vevo LAZR photoacoustic-micro-ultrasound imaging system (FUJIFILM VisualSonics, Inc., Toronto) with a 21 MHz (centre frequency) probe. The +TYR and –TYR tumors on each mouse were imaged one at a time using volumetric scans at single wavelengths, spectral scans over wavelengths from 680 to 980 nm in 5 nm increments. Significant changes were observed in photoacoustic images of the tumors after melanin induction as shown in Fig. 3. Only +TYR tumors demonstrated significantly greater photoacoustic signal after DOX administration. Since the tumor regions imaged before and after DOX treatment may be slightly different, multispectral image analysis was performed to determine the source of photoacoustic signal in the images. Multispectral and volumetric photoacoustic images were exported for offline analysis using MATLAB.

Based on the 3D ultrasound images, no significant differences were found between tumor growth rates from before and 1 week after DOX treatment (*P* = 0.86, n = 6), suggesting that +TYR and –TYR tumors have similar growth kinetics even after melanin induction (Supplementary Fig. 2). Tumor dimensions prior to DOX treatment varied greatly and +TYR tumor sizes were not statistically different from those of –TYR tumors. It is possible that melanin production may cause significant decreases in tumor growth over longer periods of time, however, the inducible tyrosinase expression system prevents cells from requiring more than 1 week under tyrosinase induced expression with DOX.

### 3D single wavelength longitudinal quantitation of photoacoustic signal

**Figure 4:**
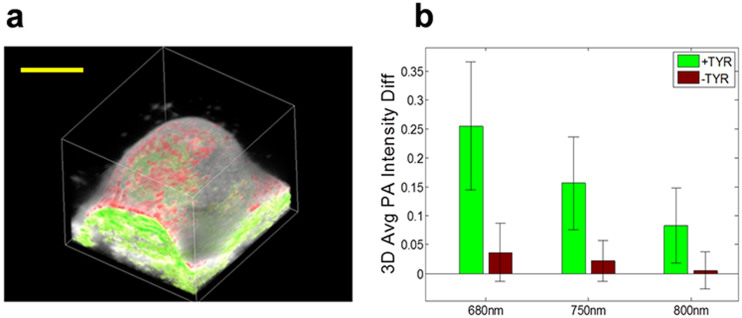
Single wavelength 3D photoacoustic and micro-ultrasound imaging. (a) Volumetric 3D visualization of +TYR tumor expressing melanin at 680 nm. Photoacoustic signal in red and green colormaps and ultrasound in grayscale. Scale bar represent 1 mm. (b) Quantification of mean photoacoustic signal difference between pre- and post-DOX treatment at wavelengths of 680, 750, and 800 nm averaged over the tumor volumes.

3D photoacoustic-ultrasound scans were acquired at a few distinct wavelengths (Fig. 4a). Ellipsoidal user-defined 3D regions of interest close to each respective tumor volume were selected in both +TYR and –TYR tumors and signal levels before and after DOX administration were quantified. Ultrasound images were used to identify the location of the surface and this was coded red whilst all regions below it were assigned green. To quantify melanin concentrations, user-selected ROIs were drawn with segmented data as a guide to avoid skin signal during tumor signal quantitation. These red- and green- photoacoustic colormaps were overlaid on grayscale ultrasound images and data was visualized using VolView visualization software. At 680 and 750 nm wavelengths (but not 800 nm), +TYR tumors demonstrated significantly increased 3D averaged photoacoustic signal compared to -TYR tumors (*P* < 0.05; Fig. 4b).

### Multiwavelength unmixing for estimation of relative melanin expression

**Figure 5:**
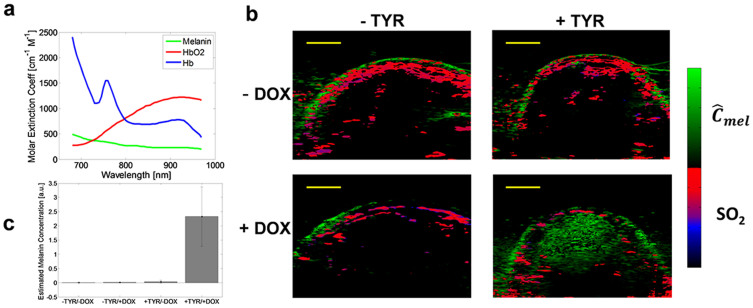
Multispectral photoacoustic imaging of -TYR and +TYR tumors. (a) Molar extinction spectra of eumelanin monomers, oxy-hemoglobin (HbO_2_) and deoxy-hemoglobin (Hb). (b) Multispectral photoacoustic imaging of -TYR and +TYR tumors. The green colormap represents estimated melanin concentration while the red-to-blue colormap is hemoglobin oxygen saturation. Scale bars represent 2 mm. (c) Quantitation of estimated relative melanin concentration levels using multispectral photoacoustic imaging.

Single wavelength photoacoustic imaging cannot allow differentiation of different sources of photoacoustic signal (e.g., oxy-hemoglobin, deoxy-hemoglobin, or melanin). To resolve the sources of photoacoustic signal, multi-spectral un-mixing was performed as described in the methods section. Multi-component in vivo photoacoustic images were generated by thresholding the total hemoglobin map (chosen as 1/3 of the maximum) and displaying *SO*_2_ estimates in pixels above this threshold in a red-blue colormap. In pixels not occupied by thresholded blood signals we displayed the melanin image in a green colormap. Images of estimated relative melanin levels in -TYR and +TYR tumors with or without DOX-treatment were segmented to select tumor tissues to avoid the skin surface and within the defined segmented regions of interest we estimated the mean (relative) melanin concentration level (Fig. 5b).

The mean tumor-melanin estimated concentrations were analyzed using 2-way ANOVA given that two key factors contributed to melanin induction: the presence or absence of tyrosinase and DOX treatment. Six mice were analyzed which had both a +TYR and –TYR tumor, and each subject was imaged before and after DOX treatment. A *P*-value of 1.2 × 10^−4^ ≪ 0.05 and 1.6 × 10^−4^ ≪ 0.05 for TYR and DOX factors, respectively, suggested that both factors were independently important for observable melanin expression. The *P*-value quantifying the interaction between the factors was estimated as 1.6 × 10^−4^ ≪ 0.05, suggesting that both DOX treatment and the presence of the inducible TYR gene interact as factors to produce strong observable melanin expression.

Non-melanin expressing tumors had estimated melanin levels which were not statistically different than each other, and the contrast-to-noise ratio of melanin-expressing tumors (+TYR/+DOX) was 430 ± 190 (Fig. 5c). Most of the variability was associated with expression level heterogeneity. The relative contrast between melanin-expressing and (-TYR/+DOX) tumors was 40 ± 17, significantly higher than was possible using a single optical wavelength where at best this contrast ratio was 7 ± 4.

Tumors as small as 1 mm were easily visualized with contrast-to-noise ratios of 190 ± 30 (Supplementary Fig. 3). Accounting for an ellipsoidal resolution voxel with the present 21 MHz transducer (~165 µm lateral resolution and ~75 µm axial resolution), and assuming ~1 billion cells per cubic centimeter of tissue, we calculate that since we can detect on the order of ~1000 cells per voxel with a contrast-to-noise of >100, we can detect mere tens of melanin-expressing cells with unity signal-to-noise ratio. Of course these detection limits will depend significantly on the amount of melanin expressed, and depth of imaging, among other factors.

### Validating sources of photoacoustic signal

**Figure 6:**
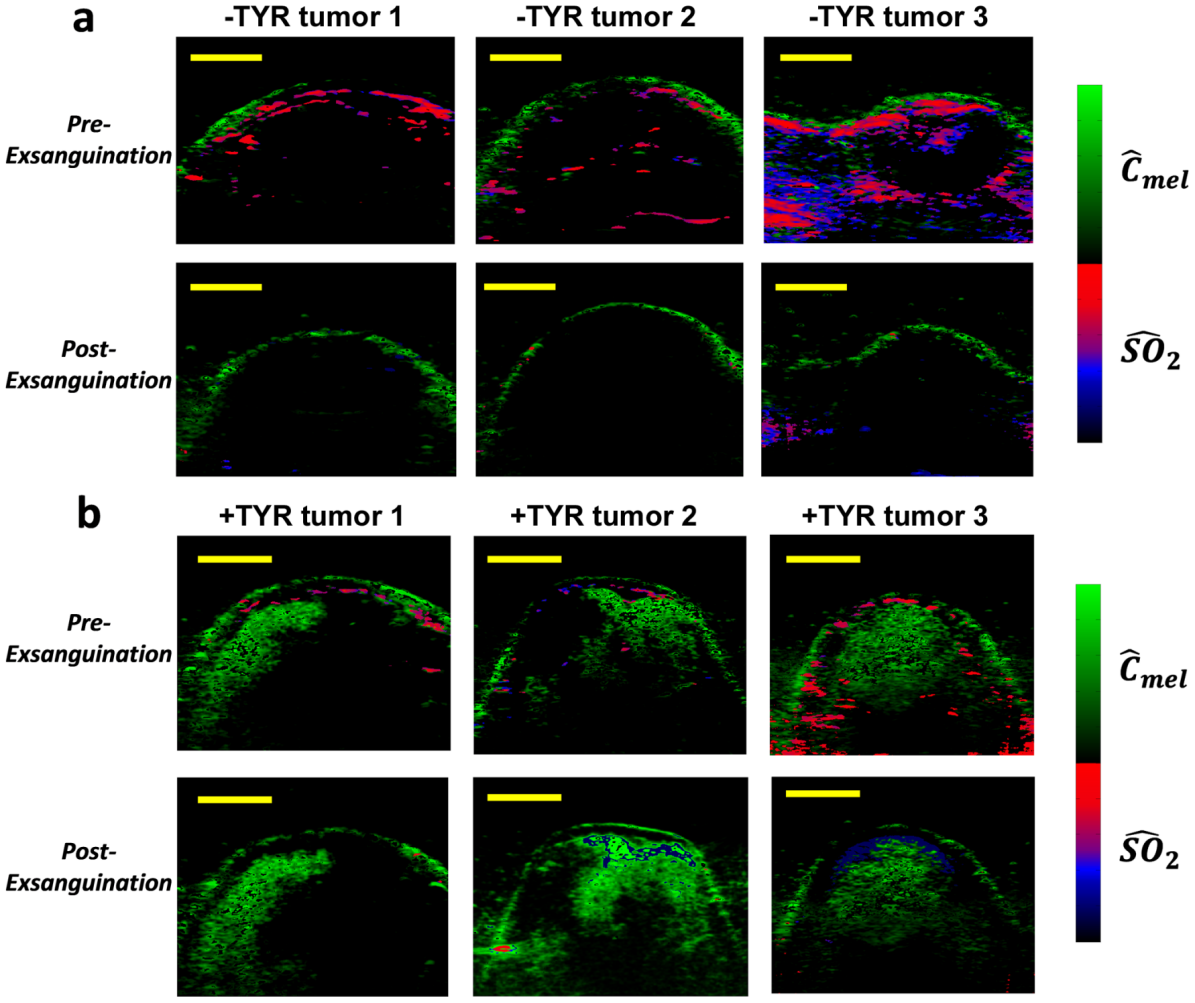
Validation of blood oxygenation and melanin signal from photoacoustic imaging. Multiwavelength images of -TYR tumors (a) and +TYR tumors (b) after in vivo DOX administration before and after exsanguination. Post-exsanguination images were acquired as close as possible to the pre-exsanguination images. Significant loss of blood is seen after exsanguination and the blood present is estimated as principally deoxygenated. Scale bars represent 2 mm.

To validate that the photoacoustic image contrast in the +TYR/+DOX tumors was principally due to melanin, we performed exsanguination to drain the animal of blood. Fig. 6 shows three –TYR tumors (Fig. 6a) and three +TYR tumors (Fig. 6b) after DOX treatment and after exsanguination. Exsanguinated control tumors exhibited very weak intratumor signals and such regions were largely classified as deoxygenated blood. In contrast, the +TYR/+DOX exsanguinated tumors exhibited strong estimated melanin signal and only weak amounts of hemoglobin signals which were principally deoxygenated. This data strongly suggests that signal in +TYR/+DOX tumor images (Figs. 3, 5, and 6) is principally due to melanin rather than hemoglobin.

**Figure 7:**
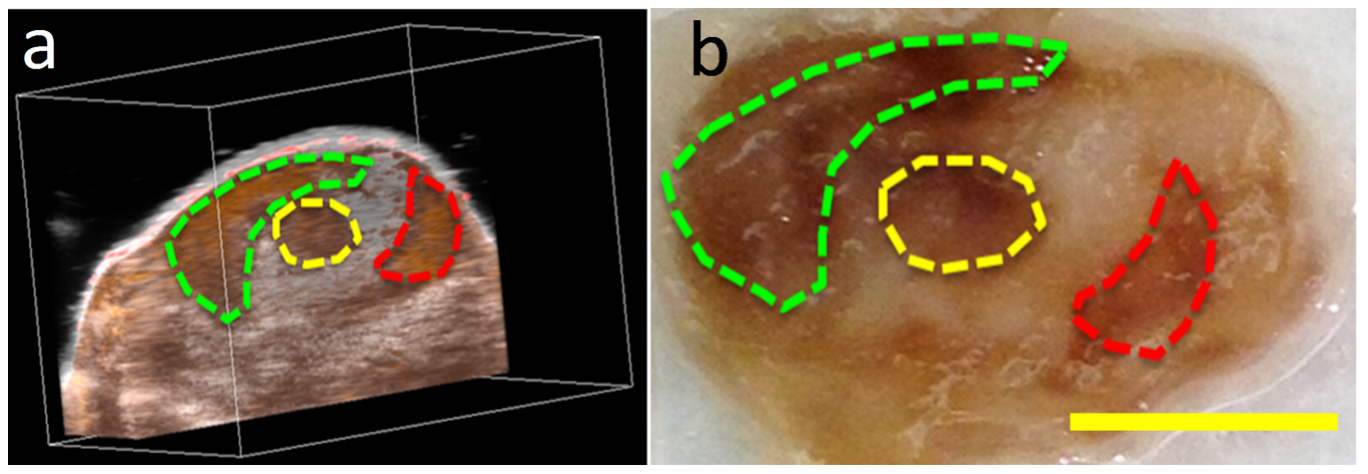
Qualitative comparison of a photoacoustic (brown colomap) and ultrasound (grayscale) image (a) and histological tumor section (b) for melanin levels in a +TYR tumor after DOX treatment. Scale bar represent 2 mm.

After all imaging sessions, tumors were excised and cut in half-mm slices parallel to the B-scan imaging plane. Photoacoustic-ultrasound images were located which closely resembled pigmentation of excised tissue sections, qualitatively confirming the ability of photoacoustic imaging to image the tumor microenvironment with high spatial resolution (Fig. 7). Both imaging and histology revealed highly heterogeneous pigmentation at a depth and resolution scale difficult to match with other optical imaging techniques. In-vivo observation of this micro-scale heterogeneity may be of significant interest to the oncologic imaging community.

### MR imaging -TYR and +TYR tumors

Melanin may be visible with MRI due to its shortening of relaxation times, and is used clinically to diagnose melanoma metastases. Since tyrosinase has previously been considered as a reporter gene for MRI^7^,^20^, we performed a study to compare the MR contrast of tumors with and without induced tyrosinase expression to the contrast of multispectral photoacoustic images. Pelleted cells and tumor-bearing mice were imaged with MRI similar to previously described methods^7^. Previously ferric citrate was used to enhance the levels of bound metals in melanin and to enhance contrast in MRI. To understand the effects of ferric citrate concentration on contrast enhancement we performed an imaging experiment involving +TYR cells with or without 1 µg/mL DOX and with varying levels of ferric citrate. Higher ferric citrate concentrations provided greater enhancements in contrast for melanin-producing cells (+DOX) compared to non-melanin producing cells (-DOX) (Supplementary Fig. 4). For in vivo MR imaging experiments, 1 mg/mL DOX was added to the drinking water of tumor bearing mice for 14 days followed by drinking water with 0.5% (w/v) ferric citrate for 4 days. Measured in vivo relative contrast of +TYR tumors compared to –TYR tumors was only 20 ± 5% with MRI (Supplementary Fig. 5) compared with nearly 19,600% for multispectral photoacoustic imaging due to the very low background of non-melanin tissues in our multispectral photoacoustic approach.

## Discussion

Photoacoustic imaging breaks resolution limits imposed by light scattering in tissues and provides an optical absorption contrast mechanism which is leveraged here to visualize blood oxygenation and inducible gene expression in tumors for the first time. These parameters are co-registered with micro-ultrasound providing a powerful toolset for preclinical research in various fields including cancer and developmental biology. Calculations indicate that photoacoustic imaging may be sensitive to only a few melanin-expressing cells. Background signal level from multi-wavelength photoacoustic imaging is significantly lower and contrast is significantly higher than that in the present MRI study.

We introduced a constrained least-squares spectral de-mixing approach which included positivity constraints and the ability to impose a minimum hemoglobin oxygen saturation (*SO*_2_) bound. When the threshold *t* was set to zero (i.e. a simple positivity constraint), lower than physiological *SO*_2_ levels were observed and may be due to known challenges for quantifying blood oxygenation using photoacoustics. Such complications include wavelength- and depth dependent fluence variations, among others. For in vivo imaging, we chose the constraint *t* to be >50% which is taken as physiologically relevant lower bound for most veins and venules. The threshold is somewhat subjective and may not always be appropriate but offers a way to incorporate a priori knowledge in the de-mixing algorithms. To validate that mean *SO*_2_ levels in live animals were significantly higher than in exsanguinated animals, the threshold *t* was set to zero for both in vivo and post-exsanguinated cases.

De-mixing algorithms used in this manuscript are not perfect and may be subject to some signal misclassification. Nevertheless exsanguination experiments validate melanin signal classification when a significant fraction of blood is removed from the animals. Skin appears to have strong melanin content even though it is visibly pale and this may in part be due to laser fluence being significantly stronger at the skin surface compared to deep tissues. It could also be a result of misclassification by the de-mixing algorithm, however there is an obvious lack of estimated melanin signal below the skin surface in control tumors thus offering confidence in subcutaneous demixing results. It should also be mentioned that motion artifacts can lead to signal misclassification. For example, the -DOX/-TYR image in Fig. 5 shows a small region coded green near a blood vessel. During realtime image acquisition this vessel exhibited pulsatile motion and thus photoacoustic images acquired using different wavelengths at different phases of pulsatility may exhibit imperfect registration leading to multi-spectral misclassification.

Although the +TYR tumors in mice treated with DOX demonstrated significant amounts of melanin production, significant heterogeneity was observed for melanin levels throughout the tumors. This may have been due to in vivo selection of cell populations with greater/lesser tyrosinase expression levels. In vitro microscopic analysis of +TYR cells treated with DOX suggests that there exists significant variability in melanin production between different cells. It is also possible that DOX from the animal drinking water was not distributed uniformly within tumors if there existed poorly perfused tumor regions. In addition, differences in tumor cell metabolism, potentially due to abnormal blood perfusion, may also affect tyrosinase expression in tumor cells. Using the tyrosinase inducible system for non-tumor models (e.g., developmental biology) may reduce the level of tyrosinase expression heterogeneity seen in tumors.

The present study validates the use of inducible tyrosinase cDNA as a photoacoustic reporter gene. The resolution (~165 µm) and depth scale (~0.5 cm) demonstrated here is sufficient to study gene expression in a variety of different animal models. Tyrosinase would be most useful for visualizing gene expression in relatively deep tissues where other optical reporters (such as fluorescent and luminescent proteins) cannot provide highly resolvable signal. Potential applications include 1) tracking tyrosinase-capable expressing cells within animal models (e.g., metastasizing cancer cells, migrating embryonic cells, genetically engineered T-cells, infectious bacterial cells, etc.) and 2) in vivo visualization of promoter activity by cloning tyrosinase downstream of promoters of interest. Future work should aim to improve quantitation and efforts are underway to quantify concentration distributions accounting for depth- and wavelength-dependent fluence and absorption-scattering non-uniqueness.

## Methods

### Cell culture and xenograft tumor creation and characterization

As previously described^7^, +TYR cells were created by stably transfecting MCF-7 Tet-On® cells (Clontech, Mountain View, CA) with a pTRE-Tight vector containing the tyrosinase gene. Using the Tet-On® system, tyrosinase expression was inducible in the presence of doxycycline (DOX). MCF-7 Tet-On® cells not stably transfected with tyrosinase were described as -TYR cells.

HEK293 and ZR-75-1 cells (American Type Culture Collection, Manassas, VA, USA), as well as MCF-7 Tet-On cells were cultured in Dulbecco's Modified Eagle's Medium (Gibco, Carlsbad, CA, USA) supplemented with 10% fetal bovine serum (Gibco, Carlsbad, CA, USA), and 200 μg/mL G-418 (A.G Scientific Inc, San Diego, CA) in T-150 cm^2^ tissue culture flasks with or without 1 μg/mL DOX (Sigma-Aldrich, St. Louis, MO, USA). After 3 days of growth, cells were trypsinized from the flasks, re-suspended in 10 mL of growth medium, centrifuged at 250 × g, washed once in phosphate buffered saline (PBS), and centrifuged to remove PBS. Cell pellets were resuspended in approximately 100 µL PBS and transferred to thin walled 200 µL PCR tubes and centrifuged to remove excess PBS. These cell pellets were used for MR imaging experiments.

All animal experiments were approved by the University of Alberta's Biosciences animal care and use committee (AUP00000061). To create xenograft tumors, at least 3 × 10^5^ -TYR and +TYR cells were suspended phenol-red-free growth medium and matrigel (1:1) and injected subcutaneously in the back left and right flanks, respectively, of 6 hairless SCID mice (Charles River, Wilmington, MA). When tumors reached at least 1 mm in diameter, mice were prepared for imaging experiments (see below). Tumor volumes in both +TYR and –TYR tumors from before to one-week after 1 mg/mL DOX drinking water treatment were imaged with photoacoustic imaging. After imaging sessions, mice were euthanized and tumors were dissected and fixed in formalin. Tumors were sectioned at half mm thickness parallel to the B-scan imaging plane and sections were photographed. These tumor slices were further sectioned to 8 µm by pathology services at the Cross Cancer Institute (Edmonton, Canada) and these sections were stained with a melanin-specific Fontana-Mason staining kit (American MasterTech, Lodi, CA, USA) according to manufacturer's instructions.

### Photoacoustic and ultrasound imaging

Ultrasound and photoacoustic imaging was performed using the Vevo LAZR photoacoustic-micro-ultrasound imaging system (FUJIFILM VisualSonics, Toronto, Canada) using a 21 MHz center frequency probe. The linear array transducer has an axial resolution of ~75 μm, lateral resolution of 165 μm, and maximum imaging depth of 20 mm. Tunable laser light is delivered downward from both long sides of the transducer head and focused at ~10 mm from the transducer head.

Phantom imaging of –TYR, +TYR cells, and blood was performed to verify tyrosinase expression in +TYR cells and validate our multi-spectral un-mixing algorithm. –TYR and +TYR cells were cultured with or without 1 μg/mL DOX for three days and were trypsinized off of the tissue culture flasks, washed once with 10 mL PBS, and resuspended at 2 × 10^7^ cells/mL in PBS. Cells were loaded into 1 mm inner diameter plastic tubing using 1 mL syringes and 18-gauge needles. Another plastic tube was filled with fresh blood (containing 4 mg/mL ethylenediaminetetraacetic acid (EDTA) as an anti-coagulant) taken from a chicken embryo. All five tubes were aligned horizontally in a custom acrylic holder, submerged in water, and imaged with the Vevo LAZR system using the 21 MHz center frequency transducer. Three-dimensional multispectral imaging was performed using 680, 700, 750, 800, 850, 900, and 950 nm laser wavelengths.

For in vivo experiments, mice were imaged before and 1 week after 1 mg/mL DOX drinking water treatment using a Vevo LAZR system with a 21 MHz and 40 MHz centre frequency probe. The +TYR and –TYR tumors on each mouse were imaged one at a time using volumetric scans at single wavelengths, and using spectral B-scan over wavelengths from 680 to 980 nm in 5 nm increments. Multispectral and volumetric photoacoustic images were exported for offline analysis using MATLAB.

### Photoacoustic multi-wavelength unmixing

Multi-spectral un-mixing was performed as follows: define a molar extinction matrix *ε* such that *ε_ij_* is the molar extinction coefficient of species 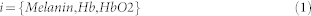
at wavelength *λ_j_*. The vector of relative concentrations to be estimated is 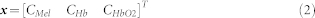
while the estimated photoacoustic initial pressure spectra at a pixel location is ***p***, where the *j* th element *p_j_* is the photoacoustic signal at wavelength *λ_j_* normalized by estimates of the laser fluence. For simplicity, we accounted for laser power variations with wavelength but not wavelength-dependent scattering and attenuation in tissue. Instead we use constrained linear least squares to estimate ***x*** for each image pixel: 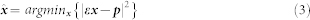
subject to constraint ***Ax*** ≤ **b**. In choosing the constraint conditions, we impose positivity of the reconstructed relative concentrations ***x*** ≥ **0**, and require the estimated oxygen saturation to be greater than some threshold *t*: SO_2_ ≥ *t* to be physiological. With these constraints it is simple to show that the constraint matrix ***A*** should be chosen as 
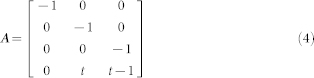
while ***b*** should be 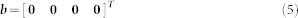
The function lsqlin in MATLAB (Mathworks Inc.) was used to implement the constrained linear least-squares unmixing. See the supplementary information for a more detailed explanation of the unmixing algorithm.

### MR imaging -TYR and +TYR cells in vitro and in vivo

Pelleted cells and tumor-bearing mice were imaged with MR as previously described^7^. Briefly, tubes or isoflurane-anesthetized mice were placed within a 44 mm inner diameter birdcage coil, tuned and matched at 400 MHz, and placed at the isocenter of a horizontal bore 9.4 Tesla animal MR imaging system (Magnex Scientific, Oxford, UK). Mice drinking water was changed to 0.5% weight/volume ferric citrate for 4 days before magnetic resonance imaging after 2 weeks of 1 mg/mL DOX drinking water treatment. Mice body temperatures were monitored via a rectal probe and maintained at 37°C using a warm air heater. Mice breathing rates were monitored by a pneumatic pillow attached just below the front ribs and breathing was maintained between 50-70 breaths per minute by varying isoflurane concentration. The quantification of T_1_ in this work was accomplished through a method similar to that used by Ponce et al.^29^.

## Author Contributions

R.J.P. created the tyrosinase expressing cells and tumors in mice, performed tumor histology, and drafted part of the manuscript. A.H. performed all photoacoustic imaging experiments and sectioned/photographed excised tumors. K.W. performed and analyzed all MRI experiments. R.J.Z. intellectually directed the project, analyzed the photoacoustic data for creation of 3D and multispectral images, as well as drafted part of the manuscript. All authors reviewed the manuscript.

## Supplementary Material

Supplementary Information Supplementary info

## References

[b1] Youn H. & Chung J. K. Reporter gene imaging. AJR Am. J. Roentgenol. 201, W206–214 (2013). 23883235 10.2214/AJR.13.10555

[b2] Min J. J. & Gambhir S. S. Molecular imaging of PET reporter gene expression. Handb. Exp. Pharmacol. 185, 277–303 (2008). 18626607 10.1007/978-3-540-77496-9_12

[b3] Zhang J., Campbell R. E., Ting A. Y. & Tsien R. Y. Creating new fluorescent probes for cell biology. Nat. Rev. Mol. Cell Biol. 3, 906–918 (2002). 12461557 10.1038/nrm976

[b4] de Almeida P. E., van Rappard J. R. & Wu J. C. In vivo bioluminescence for tracking cell fate and function. Am. J. Physiol. Heart Circ. Physiol. 301, H663–671 (2011). 21666118 10.1152/ajpheart.00337.2011 PMC3191083

[b5] Gilad A. A. *et al.* MRI reporter genes. J. Nucl. Med. 49, 1905–1908 (2008). 18997049 10.2967/jnumed.108.053520 PMC2730187

[b6] Li L., Zemp R. J., Lungu G., Stoica G. & Wang L. V. Photoacoustic imaging of lacZ gene expression in vivo. J. Biomed. Opt. 12, 020504 (2007). 17477703 10.1117/1.2717531

[b7] Paproski R. J., Forbrich A. E., Wachowicz K., Hitt M. M. & Zemp R. J. Tyrosinase as a dual reporter gene for both photoacoustic and magnetic resonance imaging. Biomed. Opt. Express 2, 771–780 (2011). 21483602 10.1364/BOE.2.000771 PMC3072120

[b8] Li C. & Wang L. V. Photoacoustic tomography and sensing in biomedicine. Phys. Med. Biol. 54, R59–97 (2009). 19724102 10.1088/0031-9155/54/19/R01 PMC2872141

[b9] Wang L. V. & Hu S. Photoacoustic tomography: in vivo imaging from organelles to organs. Science 335, 1458–1462 (2012). 22442475 10.1126/science.1216210 PMC3322413

[b10] Li L., Zhang H. F., Zemp R. J., Maslov K. & Wang L. Simultaneous imaging of a lacZ-marked tumor and microvasculature morphology in vivo by dual-wavelength photoacoustic microscopy. J. Innov. Opt. Health Sci. 1, 207–215 (2008). 19946613 10.1142/S1793545808000212 PMC2782593

[b11] Zemp R. J., Li L. & Wang L. V. [Photoacoustic imaging of gene expression in small animals *in vivo*]. Photoacoustic imaging and spectroscopy. (CRC Press, Boca Raton, 2009).

[b12] Filonov G. S. *et al.* Deep-tissue photoacoustic tomography of a genetically encoded near-infrared fluorescent probe. Angew. Chem. Int. Ed. Engl. 51, 1448–1451 (2012). 22213541 10.1002/anie.201107026 PMC3293502

[b13] Laufer J., Jathoul A., Pule M. & Beard P. In vitro characterization of genetically expressed absorbing proteins using photoacoustic spectroscopy. Biomed. Opt. Express 4, 2477–2490 (2013). 24298408 10.1364/BOE.4.002477 PMC3829541

[b14] Liu M. *et al.* In vivo three dimensional dual wavelength photoacoustic tomography imaging of the far red fluorescent protein E2-Crimson expressed in adult zebrafish. Biomed. Opt. Express 4, 1846–1855 (2013). 24156048 10.1364/BOE.4.001846 PMC3799650

[b15] Razansky D. *et al.* Multispectral opto-acoustic tomography of deep-seated fluorescent proteins in vivo. Nat. Photonics 3, 412–417 (2009).

[b16] Oetting W. S. The tyrosinase gene and oculocutaneous albinism type 1 (OCA1): A model for understanding the molecular biology of melanin formation. Pigment Cell. Res. 13, 320–325 (2000). 11041207 10.1034/j.1600-0749.2000.130503.x

[b17] Zonios G. *et al.* Melanin absorption spectroscopy: new method for noninvasive skin investigation and melanoma detection. J. Biomed. Opt. 13, 014017 (2008). 18315375 10.1117/1.2844710

[b18] Krumholz A. *et al.* Photoacoustic microscopy of tyrosinase reporter gene in vivo. J. Biomed. Opt. 16, 080503 (2011). 21895303 10.1117/1.3606568 PMC3162617

[b19] Laufer Y. *et al.* In vivo photoacoustic imaging of tyrosinase expressing tumours in mice. Proc. of SPIE, Phot. Plus Ultras. Imag. and Sens. 8223, 82230M–82231 (2012).

[b20] Qin C. *et al.* Tyrosinase as a multifunctional reporter gene for Photoacoustic/MRI/PET triple modality molecular imaging. Sci. Rep. 3, 1490 (2013). 23508226 10.1038/srep01490 PMC3603217

[b21] Stritzker J. *et al.* Vaccinia virus-mediated melanin production allows MR and optoacoustic deep tissue imaging and laser-induced thermotherapy of cancer. Proc. Natl. Acad. Sci. U S A 110, 3316–3320 (2013). 23401518 10.1073/pnas.1216916110 PMC3587225

[b22] Sze D. Y., Reid T. R. & Rose S. C. Oncolytic virotherapy. J. Vasc. Interv. Radiol. 24, 1115–1122 (2013). 23885911 10.1016/j.jvir.2013.05.040

[b23] Jordan-Sciutto K. L., Wang G., Murphey-Corb M. & Wiley C. A. Cell cycle proteins exhibit altered expression patterns in lentiviral-associated encephalitis. J. Neurosci. 22, 2185–2195 (2002). 11896158 10.1523/JNEUROSCI.22-06-02185.2002 PMC3670958

[b24] Motavaf M., Safari S., Saffari Jourshari M. & Alavian S. M. Hepatitis B virus-induced hepatocellular carcinoma: the role of the virus x protein. Acta Virol. 57, 389–396 (2013). 24294951 10.4149/av_2013_04_389

[b25] Bertram R. & Hillen W. The application of Tet repressor in prokaryotic gene regulation and expression. Microb. Biotechnol. 1, 2–16 (2008). 21261817 10.1111/j.1751-7915.2007.00001.x PMC3864427

[b26] Lee Y. B., Glover C. P., Cosgrave A. S., Bienemann A. & Uney J. B. Optimizing regulatable gene expression using adenoviral vectors. Exp. Physiol. 90, 33–37 (2005). 15542617 10.1113/expphysiol.2004.028209

[b27] Sun Y., Chen X. & Xiao D. Tetracycline-inducible expression systems: new strategies and practices in the transgenic mouse modeling. Acta Biochim. Biophys. Sin. (Shanghai) 39, 235–246 (2007). 17417678 10.1111/j.1745-7270.2007.00258.x

[b28] Alfke H. *et al.* In vitro MR imaging of regulated gene expression. Radiology 228, 488–492 (2003). 12801999 10.1148/radiol.2282012006

[b29] Ponce A. M. *et al.* Magnetic resonance imaging of temperature-sensitive liposome release: drug dose painting and antitumor effects. J. Natl. Cancer Inst. 99, 53–63 (2007). 17202113 10.1093/jnci/djk005

