# Supplementary material for: Multi-wavelength photoacoustic imaging of inducible tyrosinase reporter gene expression in xenograft tumors
Source: Sci Rep. 2014 Jun 17;4:5329. doi: 10.1038/srep05329 (PMC4060505; doi:10.1038/srep05329)
Supplement: Supplementary Information — Supplementary info [file srep05329-s1.pdf]

Multi-wavelength photoacoustic imaging of inducible tyrosinase reporter gene expression in xenograft tumors

Robert J. Paproski[1], Andrew Heinmiller[2], Keith Wachowicz[3], and Roger J. Zemp[1]*

[1]Department of Electrical and Computer Engineering, University of Alberta, Edmonton, Alberta T6G 2V4, Canada
[2]FUJIFILM VisualSonics, Inc., Toronto, Ontario M4N 3N1, Canada.
[3]Department of Oncology, University of Alberta, Edmonton, Alberta T6G 1Z2, Canada.
*Corresponding author: Email: rzemp@ualberta.ca.

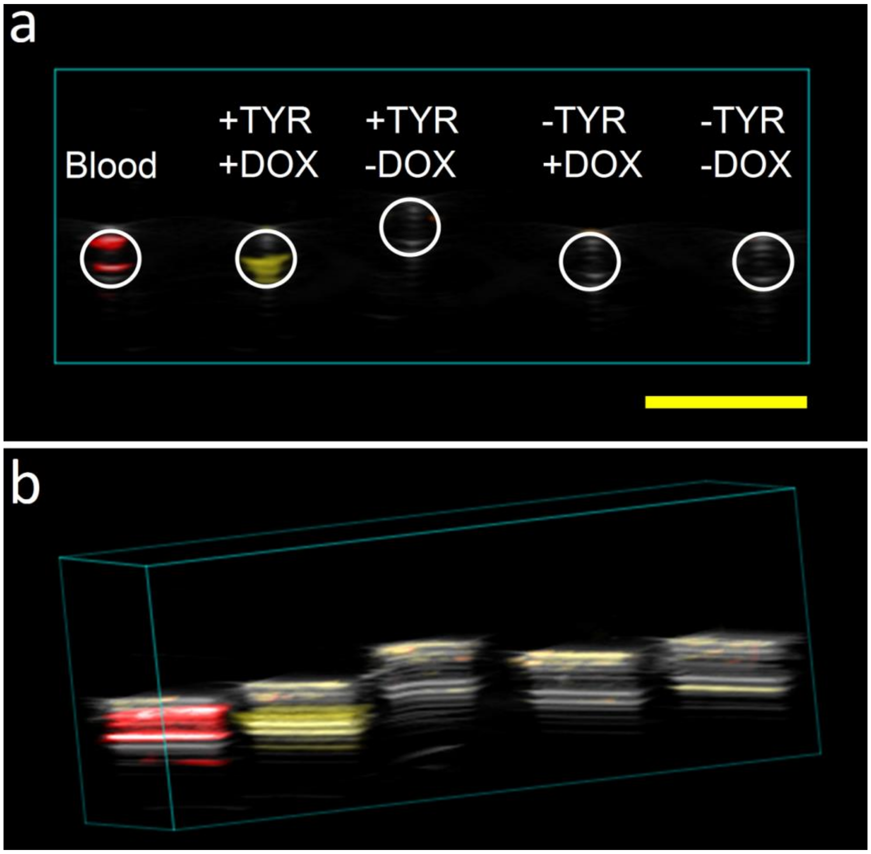

Supplementary Figure 1 Multi-wavelength 2D (**a**) and 3D (**b**) spectral unmixed photoacoustic and ultrasound image of cultured ±TYR/±DOX cells as well as blood in plastic tubes. Estimated relative melanin and hemoglobin levels are shown using yellow and red colormaps, respectively, while ultrasound is shown in grayscale. Scale bar represent 2 mm.

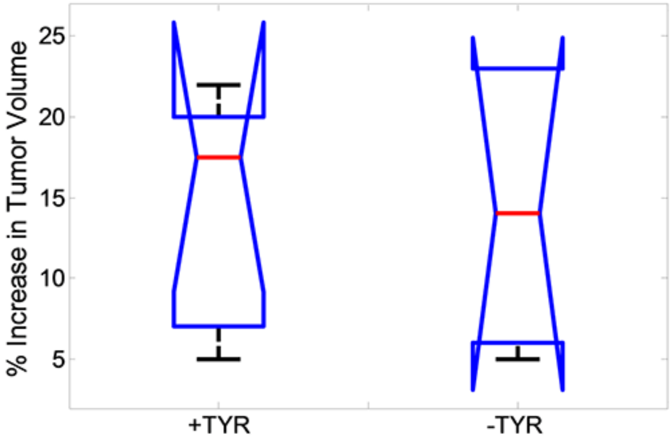

Supplementary Figure 2 ANOVA bar plot showing percent increase in tumor volumes immediately before and one week after DOX treatment.

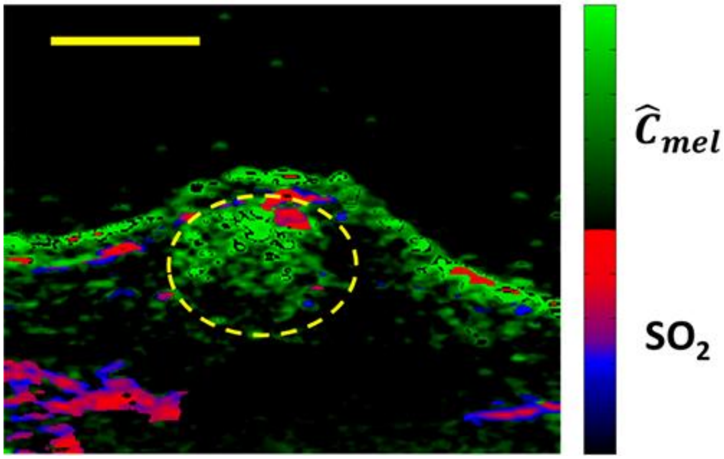

Supplementary Figure 3 Multispectral photoacoustic image of a small +TYR tumor after DOX-induction of melanin production. The scale bar represents 1 mm.

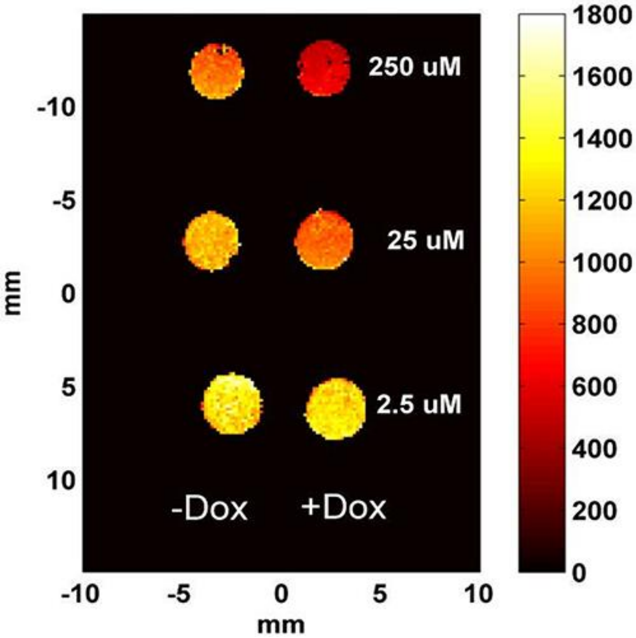

Supplementary Figure 4 Quantitative T1 relaxation times (in ms) derived from MR images of tubes containing pelleted +TYR cells. Cells were prepared with or without DOX (columns) and with varying concentrations of ferric citrate (rows).

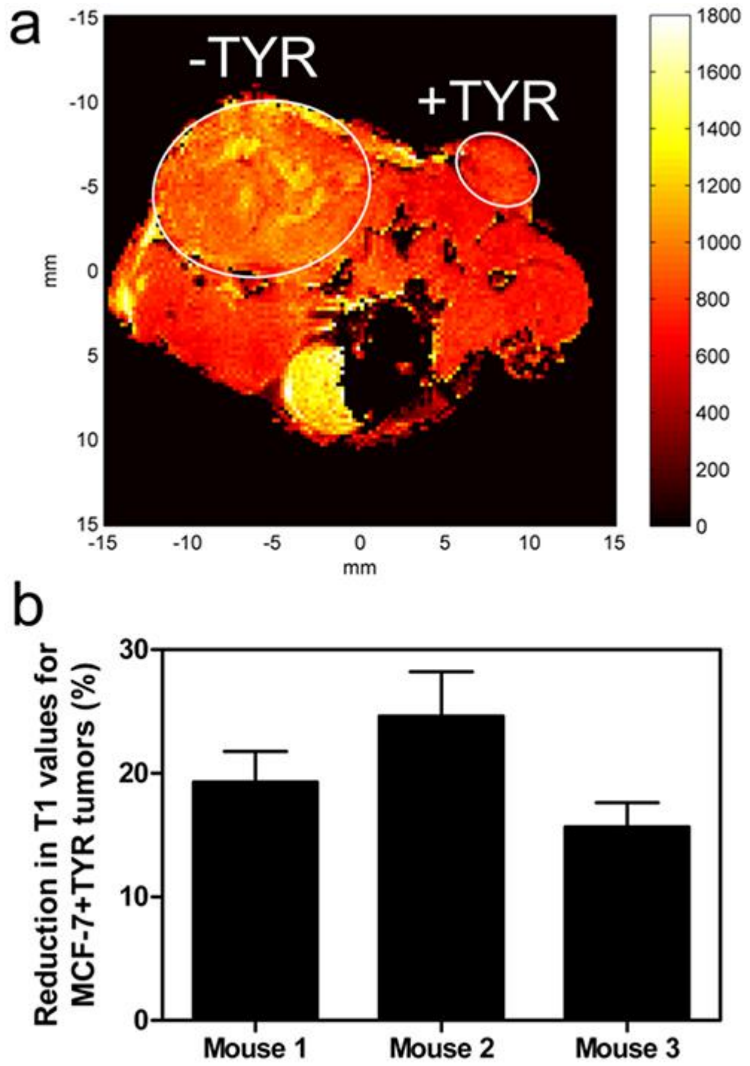

Supplementary Figure 5 In vivo MRI imaging of T1 relaxation times. (**a**) T1 map (in ms) of a mouse bearing -TYR and +TYR tumors after DOX and ferric citrate treatment. (**b**) Mean reduction in T1 values for +TYR tumors in three mice.

Supplementary explanation of multispectral unmixing algorithm

Data from multispectral photoacoustic images were de-mixed using a constrained least-squares algorithm described here. The algorithm permits positivity constraints as well as the ability to require estimated oxygen saturation levels to be above a threshold.

Define a molar extinction matrix $\boldsymbol{\varepsilon}$ such that ε_{ij} is the molar extinction coefficient of species $i = \{Melanin, Hb, HbO2\}$ at wavelength λ_j. The vector of relative concentrations to be estimated at each pixel location $\boldsymbol{r}$ is $\boldsymbol{x}(\boldsymbol{r}) = [C_{Mel}(\boldsymbol{r}) \quad C_{Hb}(\boldsymbol{r}) \quad C_{HbO2}(\boldsymbol{r})]^T$ while the estimated photoacoustic initial pressure spectra is $\widehat{\boldsymbol{p}}(\boldsymbol{r})$, where the jth element $\hat{p}_j(\boldsymbol{r})$ is the estimated photoacoustic signal at wavelength λ_j normalized by estimates of the laser fluence. We measured mean incident laser power as a function of wavelength, $\mathrm{I}_0(\lambda_j)$. The photoacoustic initial pressure at location $\boldsymbol{r}$, is modelled as $p_j(\boldsymbol{r}) = \Gamma\mu_a(\boldsymbol{r}, \lambda_j)\Phi(\boldsymbol{r}, \lambda_j)$ where $\Phi(\boldsymbol{r}, \lambda_j) \propto \mathrm{I}_0(\lambda_j)$ is the unknown wavelength dependent fluence, $\mu_a(\boldsymbol{r}, \lambda_j)$ is the local optical absorption coefficient and Γ is the Gruneisen parameter, assumed to be spatially

constant. We assume that $\mu_a(\boldsymbol{r}, \lambda_j) = \boldsymbol{\varepsilon_j x}(\boldsymbol{r})$ is a linear combination of concentrations of the dominant components (Hb, HbO_2 and melanin) weighted by the molar extinction coefficients, where $\boldsymbol{\varepsilon_j}$ is the jth column of $\boldsymbol{\varepsilon}$. Then given experimental photoacoustic images at wavelengths $\lambda_j, j = 1,2,\ldots,J$, and measured laser powers $\mathrm{I}_0(\lambda_j)$, the task is to solve the system of equations $\hat{p}_j = \alpha_j \mathrm{I}_0(\lambda_j)\boldsymbol{\varepsilon_j x} + \eta_j$ for the estimated concentrations $\hat{\boldsymbol{x}}(\boldsymbol{r})$ where η_j is noise. Here $\alpha_j(\boldsymbol{r})$ is an unknown position-dependent coefficient encompassing wavelength-dependent fluence variations and is modeled as a wavelength-independent constant α modified by a wavelength dependent perturbation $\alpha_j = \alpha(1 + \delta_j)$. In this way the system to be solved can be written as $\widehat{\boldsymbol{P}} = \boldsymbol{\varepsilon x_r} + \boldsymbol{\eta}$ where $\boldsymbol{x_r}$ are the relative concentrations (with unknown scaling constant common to all concentrations), $\widehat{\boldsymbol{P}}$ has elements $\widehat{\boldsymbol{P}}_j = \widehat{\boldsymbol{p}}_j / \mathrm{I}_0(\lambda_j)$ and are the laser-power-normalized photoacoustic signals, and $\boldsymbol{\eta}$ now encompasses both stochastic noise and bias due to perturbations δ_j. In the manuscript we refer to $\widehat{\boldsymbol{P}}$ as just $\boldsymbol{p}$ for simplicity. We use constrained least squares to estimate $\boldsymbol{x_r}$ for each image pixel. We solve

$$\hat{\boldsymbol{x}}_{\boldsymbol{r}} = \underset{\boldsymbol{x_r}}{\operatorname{argmin}} \left\{ \left| \boldsymbol{\varepsilon x_r} - \widehat{\boldsymbol{P}} \right|^2 \right\}$$

subject to constraint $\boldsymbol{A}\hat{\boldsymbol{x}}_{\boldsymbol{r}} \leq \boldsymbol{b}$. In choosing the constraint conditions, we impose positivity of the reconstructed relative concentrations $\hat{\boldsymbol{x}}_{\boldsymbol{r}} \geq \boldsymbol{0}$, and require the estimated oxygen saturation $SO_2 \equiv \frac{C_{HbO2}}{C_{HbO2} + C_{Hb}}$ to be greater than some threshold t: $SO_2 \geq t$ to be physiological. This requires $C_{HbO2}(1 - t) - tC_{Hb} \geq 0$. With these constraints it is simple to show that the constraint matrix $\boldsymbol{A}$ should be chosen as

$$\boldsymbol{A} = \begin{bmatrix} -1 & 0 & 0 \\ 0 & -1 & 0 \\ 0 & 0 & -1 \\ 0 & t & t-1 \end{bmatrix}$$

while $\boldsymbol{b}$ is taken as $\boldsymbol{b} = [\boldsymbol{0} \quad \boldsymbol{0} \quad \boldsymbol{0} \quad \boldsymbol{0}]^{\boldsymbol{T}}$. The function *lsqlin* in MATLAB (Mathworks Inc.) was used to implement the constrained linear least-squares unmixing. When $t = 0$ the function *lsqnonneg* may be used instead. In order to obtain accurate spectral de-mixing, we acquire photoacoustic images using a large number of sequential wavelengths so that the system $\widehat{\boldsymbol{P}} = \boldsymbol{\varepsilon x_r} + \boldsymbol{\eta}$ is highly over-determined, based on the hypothesis that the more non-degenerate information used, the more accurate the relative concentration estimates will be.

When displaying de-mixed images, each pixel was first classified as blood or melanin. Pixels with total hemoglobin levels greater than a threshold fraction of the maximum value (we used 50%) were classified as blood and displayed on a red-to-blue colormap representative of the estimated oxygen saturation (SO_2). The remaining pixels were assigned to a green-to-black colormap representing estimated relative melanin concentration. This estimation procedure is not rigorously quantitative, but presents a simple way to visualize approximate SO_2 and melanin distributions and observe inducible reporter gene expression in vivo.
